# The Expanding Role of Extracellular Traps in Inflammation and Autoimmunity: The New Players in Casting Dark Webs

**DOI:** 10.3390/ijms23073793

**Published:** 2022-03-30

**Authors:** Stephanie U-Shane Huang, Kim Maree O’Sullivan

**Affiliations:** Centre for Inflammatory Diseases, Department of Medicine, School of Clinical Sciences, Monash University, 246 Clayton Rd, Clayton, VIC 3168, Australia; stephanie.huang@monash.edu

**Keywords:** extracellular traps, inflammation, neutrophils, basophils, macrophage/monocytes, therapeutic targets, autoimmunity

## Abstract

The first description of a new form of neutrophil cell death distinct from that of apoptosis or necrosis was discovered in 2004 and coined neutrophil extracellular traps “(NETs)” or “NETosis”. Different stimuli for NET formation, and pathways that drive neutrophils to commit to NETosis have been elucidated in the years that followed. Critical enzymes required for NET formation have been discovered and targeted therapeutically. NET formation is no longer restricted to neutrophils but has been discovered in other innate cells: macrophages/monocytes, mast Cells, basophils, dendritic cells, and eosinophils. Furthermore, extracellular DNA can also be extruded from both B and T cells. It has become clear that although this mechanism is thought to enhance host defense by ensnaring bacteria within large webs of DNA to increase bactericidal killing capacity, it is also injurious to innocent bystander tissue. Proteases and enzymes released from extracellular traps (ETs), injure epithelial and endothelial cells perpetuating inflammation. In the context of autoimmunity, ETs release over 70 well-known autoantigens. ETs are associated with pathology in multiple diseases: lung diseases, vasculitis, autoimmune kidney diseases, atherosclerosis, rheumatoid arthritis, cancer, and psoriasis. Defining these pathways that drive ET release will provide insight into mechanisms of pathological insult and provide potential therapeutic targets.

## 1. A Brief History of the Discovery of NETs

The widely accepted inaugural characterization of neutrophil extracellular traps (NETs) is by Brinkman and colleagues in 2004. However, the first description of DNA extrusion by leukocytes dates back to 1975 by Anker et al., who showed spontaneous DNA release in vitro by viable human blood lymphocytes [1]. The authors demonstrated that these cells were capable of repeatedly releasing DNA while maintaining the ability to synthesize novel DNA without a decline in viability. Following this discovery, Rogers showed that the extruded DNA is partly composed of the newly synthesized DNA [2], and the original group found that this DNA carries the sequence to generate RNA coded for transcribing antibodies to the stimulating antigen, while spontaneously released DNA did not [3]. This suggests that these lymphocytes were capable of producing stimulant-specific DNA instantaneously to eliminate the foreign threat, providing another mechanism of innate immune defense. These were the first descriptions of a process later to be termed vital NETosis, which will be discussed in detail further on in this review.

This was followed by the characterization of suicidal NETosis by Takei et al. in 1996, whose team was studying the events between neutrophil activation and subsequent death using phorbol 12-myristate 13-acetate (PMA) [4], a robust activator of neutrophils. They observed that PMA-treated neutrophils exhibited morphological changes that were atypical of apoptosis or necrosis. They used electron microscopy to successfully describe the sequence of events involved in NET formation, starting with fusion of the lobular nucleus, followed by chromatin decondensation and nuclear envelope rupture [4]. Interestingly, the authors found that the cytoplasmic organelles had not begun degradation and membrane permeability started increasing at 3 h following PMA treatment. They also correctly hypothesized that cell death resulted from oxygen radicals as the use of antioxidants prevented PMA cytotoxicity [4].

Brinkman and colleagues [5] coined the term “neutrophil extracellular traps (NETs)” three decades after the first description of DNA release by Anker et al. [1]. Using electron and confocal microscopy, the authors were able to visualize the extrusion of fibrous structures containing DNA fibers from activated neutrophils, coated with proteins from the primary granules (neutrophil elastase, cathepsin G and myeloperoxidase), secondary granules (lactoferrin), tertiary granules (gelatinase or MMP9), and histones (H1, H2A, H2B, H3, H4, and the H2A-H2B DNA complex). The NETs were demonstrated to sequester and kill bacteria with the local delivery of antimicrobial molecules [5], the authors concluded that the sequestration of toxic granule proteins such as proteases in the fibrous structure may prevent damage to surrounding tissues. However, they also noted the release of histones may trigger the development of autoimmune conditions [5]. Since the discovery of NETs in 2004, other immune cell populations have also been characterized to release DNA structures upon stimulation. These include B cells [6,7], T cells [7,8,9], mast cells, eosinophils [10], macrophages/monocytes [11,12], basophils [13], and dendritic cells (DCs). The mechanism of DNA extrusion by these cells will be discussed later on in this review. As a variety of immune cells are capable of producing extracellular DNA traps, the mechanism will be termed ETosis to include all cell types.

While the mechanism of ETosis has been extensively studied in humans and mice since their discovery, it is a highly conserved process. ET formation has been characterized across many species, including bovine [14,15,16,17,18,19,20] porcine [21,22,23], equine [24,25,26], avian [27,28], and fish [29,30,31,32,33,34], with more recent publications adding invertebrates [35,36,37,38,39] and plants [40,41] to the repertoire. The common purpose of ETosis in these species is overwhelmingly in defense against pathogenic infection, perhaps a strategy that emerged early on in eukaryotic evolution. Using a congruency test, Ramos-Martinez et al. found that the ETs across these species exhibited convergent evolution, and the transition from being unicellular to multicellular organisms was crucial for their emergence [42]. 

As infection and immunity have been two driving forces of evolution for both host and pathogen, certain strains of bacteria have developed surface nucleases to enable their extrication from ETs [43,44,45]. For the host, circulating neutrophils are the first immune cells to be recruited to the site of infection to act as a physical barrier via the extrusion of NETs to contain the foreign pathogen; furthermore, they also play a role in wound healing. Tonello et al. showed that NETs enhanced keratinocyte proliferation in a concentration-dependent manner via a nuclear factor kappa B (NFκB) mechanism, suggesting a connection between the initial immune response and induction of wound closure [46]. NETs have also been demonstrated to induce wound healing via activation of fibroblasts in myocardial infarction patients [47], and an in vitro study found that dermal fibroblasts co-cultured with NETs upregulated α-smooth muscle actin expression and collagen production [48]. Whilst NETs can activate wound repair, they have also been shown to impair keratinocyte migration, resulting in delayed wound healing in diabetic patients and mouse models [49,50,51]. Another study showed that interaction between NETs and keratinocytes promoted Staphylococcus aureus colonization in the skin [52]. As with any immune process, appropriate activation is sufficient to return the host to homeostasis, whereas dysregulation and persistence of the inflammatory process can have detrimental outcomes. This review will focus on the mechanisms and purpose of ETosis, discuss its role in disease pathogenesis, and potential as a therapeutic target.

## 2. Pathways to NETosis

**Figure 1:**
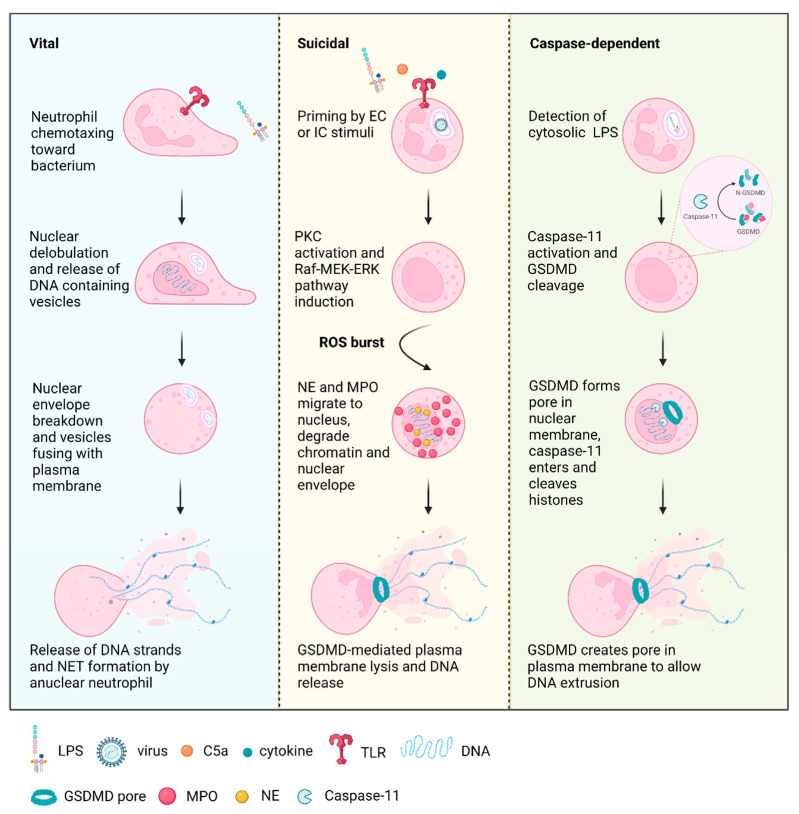
Types of neutrophil extracellular trap (NET) formation. Vital NETosis is initiated by the detection of Gram-positive or Gram-negative bacteria via Toll-like receptor 2 (TLR2) [65,66], with the neutrophil continuing to migrate toward the bacterium. This is followed by nuclear condensation and release of DNA containing vesicles, which fuse with the outer membrane and release their contents to the extracellular space to form NETs. Nuclear envelope breakdown occurs to form anuclear neutrophils. Priming of suicidal NETosis occurs with the detection of cytokines such as tumor necrosis factor α (TNFα) and interleukin 8 (IL8) [5,67]; the complement component, C5a [68]; lipopolysaccharide (LPS) [5]; and viral glycoproteins [69,70,71], among other stimulants. This activates protein kinase C (PKC) [72] and the Raf-MEK-ERK pathway [73], while the nucleus begins to lose lobules [56]. These series of events are followed by an NADPH-mediated oxidative burst [56,57], which release contents from the granules such as neutrophil elastase (NE) and myeloperoxidase (MPO) into the cytoplasm [3,74]. NE and MPO migrate to the nucleus to act synergistically for the cleavage of histones and disintegration of the nuclear membrane [74]. NE also cleaves gasdermin D (GSDMD), enabling pore formation in the plasma membrane and NET release [59]. Similar to suicidal NETosis, caspase-dependent NETosis is also dependent on GSDMD for plasma membrane pore formation. It has been found to be triggered by cytosolic LPS [75], activating caspase-11, which cleaves GSDMD, leading to pore formation in the nuclear membrane, enabling the translocation of caspase-11 into the nucleus for histone cleavage [75]. GSDMD subsequently forms pores in the plasma membrane for NET extrusion [75]. Figure created using Biorender.

NETosis is differentiated from other cell death processes such as apoptosis and necrosis by the presence of citrullinated histones, where arginine is converted to citrulline by peptidyl arginine deiminase (PAD4) [53]. The role of PAD4 in this process was further established using a pan-PAD inhibitor to demonstrate the decrease in histone citrullination, resulting in the failure of NET formation [54]. Further work from the same group used gene knockout (KO) mouse models to show the abrogation of NET-mediated bacterial killing [55]. NETosis has also been characterized to be independent of apoptotic caspases [56,57,58,59], further distinguishing it from the apoptotic process. Contradicting studies have found NETosis to be both mixed lineage kinase domain-like pseudokinase (MLKL) and receptor-interacting serine/threonine kinase 3 (RIPK3) dependent and independent using different stimuli. PMA [60], crystalline particles [61], activated platelets [62], and anti-neutrophil cytoplasmic antibodies (ANCA) were shown to induce NET formation via RIPK1 and RIPK3 [63]. Contrarily, other groups used PMA, lipopolysaccharide (LPS), or complement component C5a [64], *C. albicans*, nigericin, group B *streptococci* or the calcium ionophore A23187 [58] to show that the activation of RIPK1 or RIPK3 was not necessary to trigger NET formation. These findings suggest that NETosis may require necroptosis machinery following induction by certain stimuli but not others. A comprehensive summary of each NETosis process is shown in Table 1. This active form of cell death is characterized by two distinct phases: (1) early vital NETosis after initial phagocytosis and chemotaxis, followed by (2) late suicidal NETosis, where neutrophils release their DNA and enzymes to trap the pathogen, thus sacrificing themselves to contain the threat. These different forms of NETosis are shown in the schematic in Figure 1.

### 2.1. Vital NETosis

Neutrophils can discriminate between different bacteria and activate either vital or suicidal NETosis accordingly [76]. A dynamic process of vital NETosis has been characterized during both Gram-positive and Gram-negative infections, where neutrophils cast large NETs to prevent the dissemination of bacteria while the membrane remains intact [65,66]. This early form of vital NETosis can occur independently of reactive oxygen species (ROS) production. Platelets have been characterized to activate NETosis against Staphylococcus aureus infection [77]. Confocal and electron microscopy were used to capture the temporal events of vital NETosis in response to *Staphylococcus aureus*, showing nuclear dilatation occurring at 25 min following incubation, followed by nuclear condensation at 45 min, subsequent nuclear breakdown occurred at 60 min, with the release of DNA material into the cytoplasm [66]. Vesicles released from nuclear envelope containing DNA strands are released into the extracellular (EC) space, where they lyse and form NETs. Some dense granules were also extruded into the EC space [66].

**Table 1:** Comparisons of NETosis mechanisms.

Type of NETosis	Vital	Mitochondrial	Suicidal	Caspase Dependent
Stimuli	LPS [65,66]	GM-CSF [78], C5a [78], RNP ICs [79]	PMA [4,5,60], LPS [5], viral glycoproteins [69,70,71], *C. albicans* [80], C5a [68], IL-8 [5], crystalline particles [61], activated platelets [62], ANCA [63], TNFα [67]	Cytosolic LPS [75]
Receptors	TLR2 [65]	TLR7 [81]	TLR4 [70], TLR7 [69,71], TLR8 [69,71], C5aR1 [68], TNFαR	
Adaptors	C3 [65]		PKC [72] GSDMD [59], MLKL/RIPK3 (stimulus dependent) [60,61,62,63]	Caspase-11, GSDMD [75]
Cascades			Raf-MEK-ERK [73], ERK and p38 MAPK [70], AKT [82]	
Oxidant reliance	ROS independent [66]	ROS dependent [78,79]	ROS production [56,57,70,83]	
Components released	DNA, histones and dense granules [65,66]	Mitochondrial DNA	DNA, histones and proteins from primary [5,74,84,85], secondary and tertiary granules [5]	DNA and histones [75]
Downstream pathways activated		TLR9 [86], NFκB [87], cGAS-STING, Type I IFN [79];	Complement cascade [63], IL-17	

AKT: protein kinase B; ANCA: anti-neutrophil cytoplasmic antibody; cGAS-STING: cyclic GMP–AMP synthase and stimulator of interferon genes; EC: extracellular; DNA: deoxyribonucleic acid; ERK: extracellular signal-regulated protein kinase; GM-CSF: granulocyte-macrophage colony-stimulating factor; GSDMD: gasdermin D; IFN: interferon; IL: interleukin; LPS: lipopolysaccharide; MAPK: mitogen activated protein kinase; MEK: ERK kinase; MLKL: mixed lineage kinase domain-like; NET: neutrophil extracellular trap; NFκB: nuclear factor kappa B; PKC: protein kinase C; PMA: phorbol 12-myristate 13-acetate; Raf: rapidly accelerated fibrosarcoma proto-oncogene serine/threonine-protein kinase; RIPK3: receptor-interacting serine/threonine kinase 3; RNP IC: ribonucleoprotein immune complex; ROS: reactive oxygen species; TLR: Toll-like receptor; TNFα: tumour necrosis factor α.

Yipp and colleagues used time-lapse microscopy to study the morphological changes of neutrophils undergoing vital NETosis, demonstrating the development of diffuse decondensed nuclei that become deficient in DNA, subsequently exhibiting unusual crawling behavior where the nucleus is used as a fulcrum for crawling [65]. The study found that vital NETosis was Toll-like receptor 2 (TLR2) and complement 3 (C3) dependent, as both TlR2−/− and C3−/− mice were unable to release histones or nuclear DNA, and NETosis was restored with normal mouse serum [65]. The authors used intravital microscopy and 3D reconstruction techniques to show that the neutrophils undergoing NETosis had intact membranes and remained viable, they also continued to migrate toward bacteria [65]. This process was vital in preventing the invasion of bacteria, where disruption of NETs using DNAse resulted in bacteremia at 4 h [65]. The authors noted that as multiple bacteria have the capacity to induce DNAse and escape NETs [43,45,88,89], it is crucial for the anuclear neutrophils to maintain killing capacity. Their study found that neutrophils formed NETs while crawling and had the ability to activate phagocytosis at any time [65]. The authors also noted the ability of NETs to cause autoimmune disorders and concluded that the viable form of NETosis is tightly regulated, which may limit the bystander effect.

Recently, viable neutrophils have also been found to release mitochondrial DNA (mtDNA) NETs in a ROS-dependent manner in response to granulocyte-macrophage colony-stimulating factor (GM-CSF) and C5a [78]. Recent studies have shown the release of mtDNA NETs by low-density neutrophils in lupus nephritis, which were demonstrated to be proinflammatory and induced type I interferons (IFNs) via the cyclic GMP–AMP synthase and stimulator of interferon genes (cGAS-STING) pathway [79]. The administration of a mitochondrial ROS inhibitor was sufficient in reducing NETosis and alleviating autoimmunity in mouse models of lupus [79]. Another study showed the induction of mtDNA NET release from neutrophils following orthopedic trauma surgery [90], which induced further NET formation via TLR9 and NFκB in a nicotinamide adenine dinucleotide phosphate (NADPH) oxidase-independent manner [86,87].

### 2.2. Suicidal NETosis

The most extensively characterized form of NETosis is suicidal NET formation, which is a longer temporal process compared to vital NETosis and ends in cell death. NETs are produced by activated neutrophils, which can be stimulated by factors such as PMA, LPS, and cytokines such as interleukin 8 (IL-8) [5]. Viral infections have also been characterized to induce neutrophil activation via TLR4, 7, or 8 recognition [69,70,71]. 

The process of NET formation and release was studied by Fuchs et al. using multi-channel live-cell imaging and transmission electron microscopy [56], to characterize the event as an active form of cell death. The NETosis process starts around 60 min following PMA stimulation, where the nuclei start to lose lobules; at 80 min, the nuclei begin to expand and fill the intracellular space; 120 min is when the nuclear membrane starts to form vesicles; at 180 min, the nuclear envelope disintegrates into small vesicles and chromatin becomes decondensed; finally, at 220 min, the cells lose membrane integrity and start to rupture [56]. Several other groups have used PMA as a stimulus to characterize each of the steps in NETosis, showing the sequence of biochemical events to start with protein kinase C (PKC) activation [72]; followed by Raf-MEK-ERK pathway induction [73]; then NADPH oxidase-mediated ROS production [56,57,83]; myeloperoxidase (MPO) activation [91] and release of neutrophil proteases including neutrophil elastase (NE) and MPO from granules into the cytoplasm [74,92]; subsequent migration of NE to nucleus via a ROS-dependent process, and synergistic effect between NE and MPO drives actin degradation in an enzyme-independent process [74,92]; finally, nuclear membrane disintegration, chromatin decondensation, plasma membrane lysis, and NET release [56]. Recently, gasdermin D (GSDMD) was also found to be an essential protease in NET formation [59]. Using mutagenesis techniques, Sollberger and colleagues showed that NE released from the primary granules cleaved GSDMD at several amino acid residues similar to those recognized by caspase 4 to activate its pore-forming properties [59]. A series of inhibition experiments determined that GSDMD acted downstream of the oxidative burst [59].

Suicidal NETosis requires the production of ROS, this was demonstrated using a NADPH oxidase inhibitor, which prevented NET formation [56]. This is further supported by clinical evidence from patients with chronic granulomatous disease (CGD), who have mutations in NADPH, rendering them unable to generate ROS and subsequently unable to produce NETs [56]. However, NETosis was restored with the addition of glucose oxidase to the patients’ neutrophils, stimulating the generation of hydrogen peroxide, a ROS, resulting in the formation of NETs [56]. 

Recently, Domer and colleagues demonstrated that NETs could induce further NET formation by neutrophils, creating a positive feedback loop, amplifying bacterial killing but also inflammation if not appropriately regulated [82]. The authors also showed that NET exposure induced phosphorylation of protein kinase B (PKB or AKT), extracellular signal-regulated protein kinase 1/2 (ERK1/2) and p38; the release of IL-8 and B-cell activating factor (BAFF) by neutrophils at 18 h, but not the previously reported cytokine, tumor necrosis factor α (TNFα) [93]. This auto-amplification loop may lead to excessive release of BAFF, which may result in the generation of autoantibodies [94], leading to autoimmunity.

### 2.3. Caspase-Dependent NETosis

Cytosolic LPS or Gram-negative bacteria have been found to induce a caspase-dependent form of NETosis via the activation of caspase 11, where it cleaves GSDMD, leading to pore formation in the nuclear membrane [75]. This nuclear pore allows the entry of caspase 11 and the cleavage of histones, leading to the relaxation of chromatin. Subsequently, GSDMD creates a pore in the plasma membrane to enable the extrusion of DNA [59,75]. Surprisingly, NE, MPO, and PAD4 were not necessary in this form of NETosis, where caspase 11 and GSDMD are the direct inducers [75]. 

**Figure 2:**
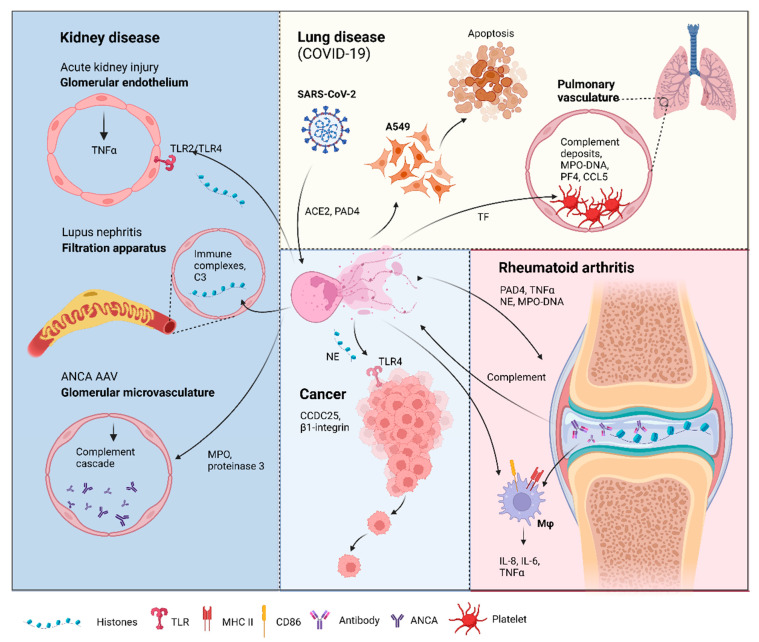
Neutrophil extracellular traps (NETs) in disease pathology. Acute kidney injury: NETosis in the glomerular capillaries results in histone release, triggering renal cell necrosis via Toll-like receptor 2 (TLR2)/TLR4, leading to tumor necrosis factor (TNFα) release [105]. Lupus nephritis: Accumulation of immune complex deposits containing anti-double-stranded DNA (dsDNA) IgG and its target, plus the complement component, C3, attracts neutrophils to the renal filtration apparatus [106]. Presence of hyperacetylated histones in the immune deposits trigger an increase in reactive oxygen species-independent NETosis [107]. Anti-neutrophil cytoplasmic antibodies (ANCA)-associated vasculitis (AAV): Neutrophils from AAV patients undergo spontaneous NETosis [108]. ANCA against myeloperoxidase and proteinase 3, two proteins in neutrophil granules are found in the glomerular microvasculature 135 [109]. These autoantigens activate the complement cascade and induce further NETosis [110,111]. Lung disease in coronavirus disease-19 (COVID-19): Severe acute respiratory syndrome-related coronavirus 2 (SARS-CoV-2) can induce NETs via an angiotensin-converting enzyme 2 (ACE2) and PAD4-dependent mechanism [112]. These NETs can enhance the apoptosis of a human alveolar basal epithelial cell line, A549 [79]. Complement deposits in the pulmonary vasculature prime neutrophils for NETosis, resulting in myeloperoxidase (MPO)-DNA complexes. Elevation of platelet factor 4 (PF4), chemokine ligand 5 (CCL5) [113], and the neutrophil–platelet interaction all trigger NETosis [114,115], which was shown to have thrombogenic activity via the upregulation of tissue factor (TF) [114]. Rheumatoid arthritis (RA): Peptidyl arginine deiminase 4 (PAD4) citrullinates histones released by spontaneous NETosis, forming immune complexes in the synovial joint that activate macrophages (mφ) [116]. The immune complexes can also activate the complement cascade, which triggers, further NETosis [117]. NETs from RA donors can activate resting macrophages to induce the surface expression of major histocompatibility complex II (MHCII) and cluster of differentiation 86 (CD86) [93]. These macrophages also release interleukin-6 (IL-6), IL-8, and TNFα [93], which can feedback to induce more NET formation. Cancer: Cancer cells attract neutrophils into the cancer microenvironment (CME), which results in NET formation under the inflammatory CME. These NETs can capture circulating tumor cells via β1-integrin [101], citrullinated histones from NETs can also enhance cancer metastasis by binding to coiled-coil domain-containing protein 25 (CCDC25) [103]. NETs and the neutrophil elastase (NE) released can promote tumor cell growth via TLR4 [118]. Figure created in Biorender.

While the different mechanisms of NETosis create a comprehensive repertoire in the prevention of pathogenic invasion, it also has the ability to cause excessive and chronic inflammation, with the potential for the development of autoimmune conditions, a few of which will be discussed in this review. An overview of the involvement of NETs in the pathogenesis of each disease is summarized in Figure 2.

## 3. NETs—Friend or Foe in Human Disease?

### 3.1. Cancer

Cancer cells attract neutrophils (tumor-associated neutrophils, TANs), which result in the release of NETs into the tumor microenvironment. NETs have been found in several types of mammalian tumors, including pancreatic, breast, liver, lung, and gastric cancers; their presence in solid tumors is associated with poorer prognosis [95,96,97]. While NETs can exert anti-tumor actions via MPO [98], proteinases [95,99], and histones [100], the proteinases can also digest the extracellular matrix (ECM), enhancing metastasis [95,99]. Additionally, it has been shown that NETs are capable of capturing circulating tumor cells via the upregulation of β1-integrin expression, an interaction that was abrogated using DNase [101]. Therefore, it is unsurprising that NETs have been found around metastatic tumors [102]. In fact, citrullinated histones from NETs were found to promote cancer metastasis by binding to coiled-coil domain-containing protein 25 (CCDC25) [103]. CCDC25 knockout cells were found to lose their adhesive properties to NET-DNA; NET-DNA-induced cytoskeleton remodeling was inhibited; and chemotaxis toward NET-DNA was reduced [103]. Injection of the CCDC25 KO cells into immunocompromised mice significantly inhibited NET-mediated lung metastases. Contrarily, overexpression of CCDC25 enhanced cancer cell adhesion and migration toward NET-DNA in vitro, and promoted the formation of lung and liver metastases in vivo [103]. Additionally, the authors found high CCDC25 expression in primary tumors to be associated with poor prognosis [103]. Another group showed the ability of NETs to promote gastric cancer metastasis via the induction of epithelial–mesenchymal transition (EMT), enabling intravasation of tumor cells into the circulation or lymphatics, leading to tumor formation at a distant tissue site (Pastushenko, 2019). Using Western blot and immunohistochemical analysis, the authors showed decreased levels of E-cadherin, an epithelial adhesion marker, and increased levels of vimentin, a mesenchymal marker in human gastric adenocarcinoma cells treated with NETs [104].

In addition to the pro-metastatic function of NETs, they have also been shown to promote tumor cell growth by enhancing their mitochondrial functions [118]. Yazdani et al. showed that stressed cancer cells upregulate the expression of several chemokines and high-mobility group box 1 (HMGB1), recruiting neutrophils to the tumor microenvironment (TME), inducing NET formation. NETs and the NE present in the fibrous structure were demonstrated to promote mitochondrial homeostasis, increasing energy production, and accelerating cancer cell proliferation via TLR4. The blockade of this interaction through genetic manipulation, pharmaceutical inhibition or siRNA targeting of the axis components were shown to abrogate mitochondrial biogenesis and impede tumor growth [118].

### 3.2. Multiple Sclerosis

Myelin damage in the white matter may result from the failure to maintain compact myelin, resulting from abnormally enhanced citrullination of myelin basic protein (MBP), which form less cationic isomers, thereby destabilizing the myelin multilayers [119]. This also enhances the susceptibility of the myelin sheath to digestion by proteases [120]. Additionally, citrullinated MBP causes fragmentation of lipid vesicles of the myelin layer, which may contribute to further demyelination [121,122]. Furthermore, studies have found the degree of MBP citrullination to correlate with disease severity [123,124]. Unsurprisingly, increased levels and enzyme activity of PAD4 have been found in the central nervous system (CNS) of multiple sclerosis (MS) patients and animal models, with an associated increase in citrullinated histone observed in the white matter [54,125]. Nuclear PAD4 and TNFα were shown to be elevated before the onset of the demyelinating disease in mouse models [83], suggesting their value as prognostic markers for MS. Furthermore, an elevated level of TNFα was detected to be released by astrocytes and not infiltrating immune cells [125], which may contribute further to local inflammation by priming neutrophils for NET formation. 

Current clinical evidence suggests that neutrophils may contribute to MS pathogenesis, as they are not only found in increased frequency in MS patients, but the neutrophils are also in an activated state with a reduction in apoptosis. These neutrophils also have increased levels of TLR2, IL8R, enhanced degranulation, and oxidative burst [126]. Elevated levels of NETs have been detected in the serum of MS patients [126], with NE levels directly correlating with disease severity and clinical prognosis [127], while significantly more MPO-DNA were complexes detected in male patients, who tend to have poorer prognosis than their female counterparts [126,128]. Pharmaceutical intervention may also play a role in disease progression, where patients on corticosteroid treatment were found to have significantly increased NE activity, whereas patients on IFNβ-1b treatment showed a lower level of NE and immune complexes [129]. 

The most recent elucidation of the mechanistic role of NETs in MS pathogenesis was performed by Wilson et al., showing that histones can directly activate T cells for T-helper 17 (Th17) differentiation via TLR2 and myeloid differentiation primary response 88 (MyD88), leading to the phosphorylation of signal transducer and activator of transcription (STAT3) [95], essential for Th17 cell responses [96]. Komiyama et al. also demonstrated the ability of NETs to activate inflammatory Th17 cells, which can secrete IL-17, a neutrophil chemotaxis cytokine [94]. Using IL-17−/− mice, the authors showed a significant decrease in immune cell infiltrate, delayed the onset and reduced disease severity [94], providing a potential therapeutic target for MS treatment. Other groups have demonstrated that pharmaceutical inhibition of MPO reduced oxidative stress and enhanced integrity of the blood–brain barrier (BBB), decreasing disease severity [130,131].

### 3.3. Kidney Disease

The kidney is prone to immune responses that lead to the development of renal injury and chronic kidney disease progression [132,133,134,135]. This can trigger neutrophil infiltration to the site of injury, compounded by the damage-associated molecular patterns (DAMPs) released by the necrotic renal cells, inflammation and tissue damage become amplified in the kidney [133,136]. The released DAMPs then go on to stimulate the infiltrating neutrophils and induce PAD4 activation, which triggers chromatin decondensation and NET formation [137].

#### 3.3.1. Acute Kidney Injury (AKI)

Acute kidney injury can occur as a consequence of necroinflammation, an interplay of injury and inflammation, where complement activation, exotoxins, or cytotoxic T cells can trigger renal cell necrosis [136]. Kumar et al. showed that NETosis results in histone release, leading to glomerular endothelial cell death in an TLR2/TLR4-dependent manner [105]. EC histones also trigger the release of TNFα in the glomerular capillaries [105], a cytokine that is not only an inducer of NET formation [93], but also characterized to be prothrombotic [138,139,140]. This study provided evidence for the presence of fibrinogen within the glomerular capillaries, which was abrogated with an anti-histone IgG [105]. Damage by histone release from NETosis was further characterized by Nakazawa et al. to show that histone citrullination is essential in inducing NET formation [137]. Additionally, tubular cells killed via NETosis released factors that triggered further NET formation [137], creating an auto-amplification loop for inflammation.

#### 3.3.2. Lupus Nephritis

NETosis is commonly found in active systemic lupus erythematosus (SLE) [106]. One of the most serious complications of SLE is nephritis, inflammation of the renal filtration apparatus, with chronic autoimmune insults to the glomerulus eventually leads to kidney failure [141,142,143]. A key characteristic of lupus nephritis is immune complex deposits containing anti-double-stranded DNA (dsDNA) IgG and its target, along with C3 accumulation, both attracting inflammatory cell infiltrates [106]. Furthermore, the presence of dsDNA can activate type I IFN signaling via the cGAS-STING pathway [144], enhancing the inflammatory response.

Presence of NETs in renal biopsies from lupus nephritis patients may indicate their contribution to kidney damage [145]. Rother et al. revealed that hyperacetylated histones were present in the SLE immune deposits and stimulated an increase in ROS-independent NETosis within 30 min [107]. They also showed that immune deposits from patients with active lupus nephritis displayed the highest acetylation levels and were the most potent at inducing NETs compared to SLE patients without nephritis [107]. Furthermore, SLE patients with nephritis or prior nephritis have an inability to degrade NETs due to impaired DNase activity [107]; thus, existing NET deposits can stimulate further inflammation. This impaired NET removal from patient serum correlates with proteinuria, decreased albumin levels, and lower creatinine clearance rates, all indicators of active nephritis [146,147].

#### 3.3.3. ANCA-Associated Vasculitis

ANCA-associated vasculitis (AAV) comprises part of a group of immune vasculitides, characterized by necrotizing inflammation of small vessels and circulating ANCAs [148]. The three types of AAV include granulomatosis with polyangiitis (GPA), microscopic polyangiitis (MPA), and eosinophilic granulomatosis with polyangiitis (EGPA) [149]. The presence of ANCA against myeloperoxidase (MPO) and proteinase 3, two proteins in neutrophil granules, is characteristic of ANCA-associated vasculitis [109]. As discussed previously, both autoantigens are present in NETs, and have been characterized to increase cell adhesion, activate the complement cascade, and induce NET production [110,111]. MPO is recognized as an autoantigen by MPO-specific autoantibody, resulting in a vicious cycle of inflammation where the generation of circulating ANCAs activate neutrophils which deposit MPO in the glomeruli and recruit macrophages and CD4 T resulting in inflammation and the induction of glomerulonephritis. This manifests clinically as MPA [150], an ANCA-associated pathology that affects the small vessels in the renal glomeruli in particular, but can also effect the blood vessels in the lungs and skin [151]. Immunoglobins purified from MPA patients have demonstrated the ability to induce NETs in direct correlation with the affinity of ANCA for MPO [150]. DNAse I activity is also lower in MPA patients, indicating insufficient degradation of NETs [152], which has been found to be responsible for the renal damage observed in up to 90% of MPA patients [108].

Although the current paradigm assumes that ANCAs induce NET formation, neutrophils from AAV patients are not prone to apoptosis, but can spontaneously undergo NETosis [108]. Furthermore, recent data have shown that NETosis in AAV is independent of serum ANCA levels [153]. Additionally, excessive NET formation in AAV is directly correlated with active clinical disease rather than severe infection, implicating the significant role of NETs in autoimmunity [153].

#### 3.3.4. Goodpastures Disease (Anti-Glomerular Basement Membrane Disease)

Akin to lupus nephritis and ANCA-AAV, the pathogenesis of anti-glomerular basement membrane (GBM) disease involves both immune complex deposition and microvascular damage [154]. These immune complexes stimulate NETosis, triggering FcγR-mediated endocytosis and tyrosine-protein kinase ABL/proto-oncogene tyrosine-protein kinase Src (Abl/Src) kinase activation, leading to glomerular endothelial injury [155,156], where disease could be attenuated by inhibiting Abl/Src signalling in a bovine model [156]. A murine anti-GBM glomerulonephritis model showed the presence of NETs in a necrotising lesion, where histones from NETs were shown to cause capillary injury, which was ameliorated using PAD inhibitors and anti-histone antibodies [105]. Contrarily, research by another group found that PAD4 inhibition using either antibody or genetic deletion had no protective effect in anti-GBM disease [157], challenging the role of NETs in anti-GBM pathogenesis. 

#### 3.3.5. Vasculitis

Vasculitis is a broad term encompassing diseases involving inflammation of the blood vessels [158], it includes ANCA-associated vasculitis (AAV, small-vessel vasculitis), Takayasu arteritis (large-vessel vasculitis), and giant cell arteritis [159]. Contention exists around whether NETosis is a hallmark of active AAV as high levels of NET remnants are found in all three types of vasculitis mentioned above [108]. However, proteinase 3-ANCA (PR3-ANCA) and MPO-ANCA have been found in most vasculitis patients, both characteristic of AAV pathogenesis [160]. Several studies have shown that autoantibodies, serum immune complexes, and IgG derived from patients with AAV have the capacity to induce NETosis [145,152,161,162,163,164]. Recent work has shown that MPO-ANCA may cause NETosis in patients with vasculitis due to its affinity rather than the antibody levels [152,162,165]. These findings are further supported by a renal biopsy study, where individuals with high-affinity MPO-ANCA demonstrated enhanced levels of NETosis [153]. 

PR3-ANCA and MPO-ANCA can cause NET formation in patients with AAV [152]; however, the mechanism by which this occurs remains unclear. As discussed previously in this review, NETosis requires neutrophil activation, an event characterized by the respiratory burst, which was induced by crosslinking PR3-ANCA or MPO-ANCA to the cell surface [166]. Furthermore, many factors released by neutrophils can activate the complement cascade [167,168,169], and several animal studies have shown that C5a–C5aR interactions can prime neutrophils for activation by ANCA [68,111,170]. C5aR1 may be a promising therapeutic target, as inhibition with antagonist CCX168 (Avacopan) has demonstrated efficacy in mediating disease severity [170]. Other autoimmune studies have found that NETosis may be negatively regulated by the interaction between neutrophil semophorin D and plexin B2 on endothelial cells, this is further evidenced by the ability of recombinant plexin B2 in the blockade of NETosis in human cells.

### 3.4. Rheumatoid Arthritis

**Figure 3:**
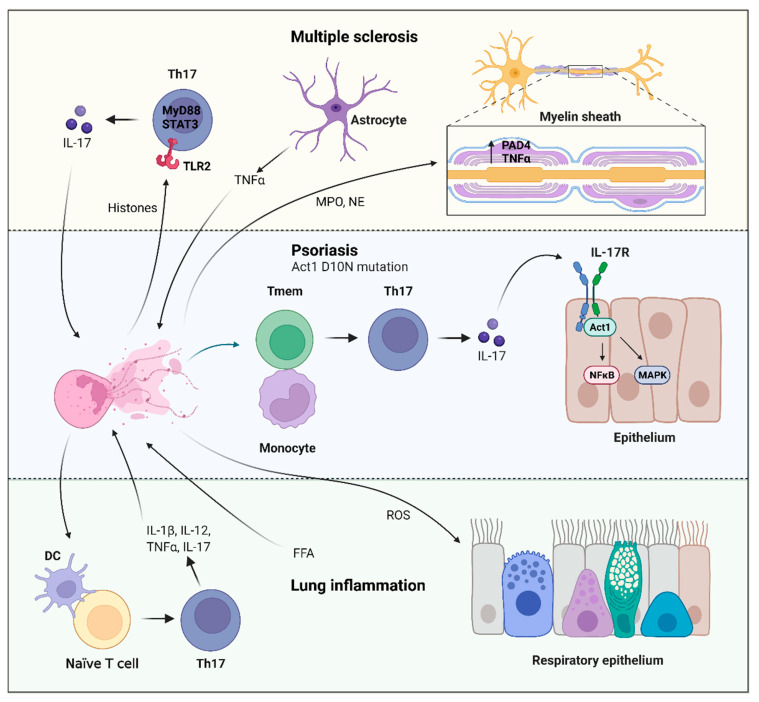
Neutrophil extracellular trap (NET) induction of T helper 17 (Th17) cells in disease. Multiple sclerosis (MS): Histones released from NETosis can directly trigger Th17 differentiation via Toll-like receptor 2 (TLR2) and myeloid differentiation primary response 88 (MyD88), activating signal transducer and activator of transcription (STAT3), which is critical for Th17 function [204,205]. The secretion of interleukin-17 (IL-17) from the Th17 cells recruit more neutrophils [206], creating a proinflammatory feedback loop. Astrocytes can also release tumor necrosis factor α (TNFα), which can prime neutrophils for NETosis [125], contributing to further local inflammation. Elevated myeloperoxidase (MPO) and neutrophil elastase (NE)-DNA immune complexes have been shown to correlate with MS disease severity [126,127,128,129]. Elevated nuclear levels peptidyl arginine deiminase 4 (PAD4) and TNFα were detected before demyelinating disease. Psoriasis: Loss of function mutation in Act1, D10N, has been associated with disease susceptibility [193,194,195,196]. With these individuals spontaneously generating NETs, which trigger memory T cells (Tmem) that are co-cultured with monocytes to differentiate into Th17 [200]. The IL-17 secreted by the Th17 cells bind to interleukin-17 receptor (IL-17R) and activates the ubiquitin adaptor Act1 (NFκB activator 1), subsequently triggering the nuclear factor κ B (NFκB) and mitogen-activated protein kinase (MAPK) pathways in target cells such as keratinocytes, epithelial cells, and fibroblasts [190,191,192]. Lung inflammation: Free fatty acids (FFAs) can induce NET release, leading to acute pulmonary injury via ROS production [201]. These NETs can also activate dendritic cells (DCs), which can trigger the differentiation of naïve T cells into Th17 cells, releasing IL-1β, IL-12, and TNFα [201,203]. Figure created using Biorender.5.2 T cells.

As discussed earlier in this review, NETosis is distinguished from other forms of cell death by the presence of citrullinated histones, a PAD4-mediated process. The involvement of PAD4 in the citrullination of histones was uncovered in animal models of rheumatoid arthritis (RA), where the presence of PAD4 and citrullinated proteins showed a positive correlation with disease progression [171]. RA is characterized by the generation of autoantibodies against citrullinated proteins, which form immune complexes with citrullinated fibrinogen in the synovial tissue, resulting in the activation of macrophages [116]. As citrullinated histones are found within NETs, as mentioned earlier on in this review, neutrophils undergoing NETosis may also become a target; therefore, any areas of local inflammation can become amplified with autoantibody binding and further exacerbation of injury. This process can become a self-perpetuating cycle, where autoantibodies targeting the citrullinated histones in NETs can also activate the complement system [117], releasing products from the complement cascade that prime neutrophils for NETosis, as discussed previously. This may provide an explanation for the accepted paradigm where complement activation at the site of autoantibody binding of citrullinated proteins can result in tissue damage [172] (Figure 3).

NETs from RA patients induce stronger inflammatory response compared to NETs from healthy donors [93]. Furthermore, neutrophils from RA patients showed increased spontaneous NET formation in vitro, with potential for elevated ROS production, increased NE and MPO expression, PAD4-mediated citrullination of H3. This was all abrogated with IgG depletion, but restored with an anti-citrullinated peptide antibody [173]. Additionally, NETs from both healthy and RA donors were shown to activate resting macrophages, inducing the upregulation of human leukocyte antigen (MHC) class I/II and cluster of differentiation 86 (CD86) on the cell surface [93]. Furthermore, NET stimulation of the resting macrophages induced the secretion of IL-8, IL-6, and TNFα [93], all proinflammatory cytokines that have been characterized to be dysregulated in RA and other autoimmune conditions [174,175,176,177], where antibodies targeting the latter two cytokines are currently used in the clinic for the treatment of RA [177,178]. Despite the pathogenic role of NETs in RA, Ribon and colleagues showed that they have anti-inflammatory effects on LPS-activated macrophages, inhibiting the release of IL-6 [93]. The authors concluded that NETs can trigger both pro- or anti-inflammatory effects depending on the target cell, its activation status, and the stimulus [93].

### 3.5. Lung Disease

While NETs in the pathogenesis of lung disease have been extensively characterized, recent research has provided evidence for their involvement in severe acute respiratory syndrome-related coronavirus (SARS-CoV-2) infection. A higher abundance of circulating neutrophils has been correlated with a worse clinical outcome in coronavirus disease 2019 (COVID-19) [179,180]. As NETosis is an important effector function of neutrophils under inflammatory conditions, it was not surprising that MPO and histone 3 (H3), both NET components, levels were found to be elevated in the plasma, tracheal aspirate, and lung biopsies from COVID-19 patients [112,181]. Their neutrophils were capable of extruding more NETs, which were also larger than those produced by neutrophils from healthy controls [112]. SARS-CoV-2 induction of NETs is dependent on angiotensin-converting enzyme 2 (ACE2), serine protease, viral replication, and PAD4 [112]. The release of NETs from the SARS-CoV-2-activated neutrophils was found to enhance the apoptosis of A549 cells, a human alveolar basal epithelial cell line [112], which can compromise lung function and lead to further disease progression. 

Thrombogenic events have been documented in severe COVID-19 patients to induce lung damage [182]. Significant deposits of complement components have been found in the pulmonary microvasculature of severe COVID-19 patients with respiratory failure [182]. As discussed previously, the presence of complement elements can prime neutrophils for NETosis, with the addition of the cytokine storm observed in these patients [183], another NET inducer, it may not be unreasonable to assume that NETosis is contributing to microvasculature damage. This has been evidenced by a recent study, where NETs were found to colocalized with platelets in pulmonary vasculature from COVID-19 autopsies and disease severity correlated directly with MPO-DNA complexes [113]. Furthermore, there was a significant elevation of platelet-derived factors such as platelet factor 4 (PF4) and chemokine ligand 5 (CCL5 or RANTES) that activate NET formation [113]. Another study characterized the interaction between COVID-19 and C5aR1, where MPO-DNA complex levels were found to correlate with thrombin/antithrombin (TAT) activity, suggesting activation of the thrombin axis [184]. This neutrophil–platelet interaction is necessary for NET formation in thromboinflammatory disorders [114,115], their presence was detected in aggregates in the circulation by Middleton et al. [113]. Additionally, NETs have demonstrated thrombogenic activity in many inflammatory disorders via the expression of functionally active tissue factor (TF) [114]. Skendros et al. found that platelet-rich plasma-stimulated neutrophils induced the expression of TF mRNA, efficiently produced NETs bearing TF, which showed high TAT activity [184]. As C5a is key mediator of neutrophil TF expression [185,186,187], Skendros and colleagues showed that selective blockade attenuated TF expression and functionality, inhibiting NET release [184].

## 4. Role of NETs in Inducing T Cell Responses 

NETs are able to induce Th17 differentiation in a TLR2/MyD88-dependent manner. The phosphorylation of STAT3 is a crucial event in the activation of Th17 responses. As Th17 activation by NETs has been described extensively in psoriasis and pulmonary inflammation, the data from these studies will be utilized to shed light on the possible mechanisms that may be involved.

Psoriasis susceptibility genes have been identified as tyrosine kinase 2 (TYK2) and TRAF3-interacting protein 2 (TRAF3IP2). Additionally, NETs promote Th17 induction, leading to IL-17 production, which binds to the IL17 receptor, activating the ubiquitin adaptor, Act1 (NFκB activator 1) [188,189,190]. This subsequently activates the NFκB and mitogen-activated protein kinase (MAPK) pathways in target cells such as keratinocytes, epithelial cells, and fibroblasts [190,191,192]. A loss-of-function mutation in Act1, D10N, leading to the replacement of an aspartic acid (D) with asparagine (N), predisposes individuals to psoriasis [193,194,195,196]. Wang et al. showed that Act1 is a client protein of the molecular chaperone, heat shock protein 90 (Hsp90). Subsequently, using Act−/− mouse models, they demonstrated that the Act1 D10N single nucleotide polymorphism (SNP) is defective, thereby leading to the loss of Act1 function [197]. They went on to demonstrate a hyper Th17 response in Act−/− mice, with spontaneous development of IL-22-mediated dermatitis, providing a potential mechanism for the Act1 D10N mutation in psoriasis susceptibility [197].

NETs have been characterized to contribute to the overactive Th17 response in psoriasis pathology, with elevated levels found in the blood and lesions [198,199]. Lambert et al. used depletion experiments to investigate the role of NETs in the induction of Th17, showing that memory T cells rather than naϊve T cells was the population from which the Th17 cells were derived [200]. They went on to show that the Th17 phenotype was only induced when the memory T cells were in physical contact with monocytes. Using donors with the Act1 D10N variant that produced spontaneous NETs, they showed that IL-17 release was more prominent in the homozygote genotype compared to wild-type homozygotes. Furthermore, global gene expression analysis revealed a stimulatory effect of NETs on the Act1 D10N genotype, with enrichment of the “cytokine activity” gene set [200].

In pulmonary inflammation, the presence of free fatty acids (FFAs) has been characterized to induce NET release, resulting in acute lung injury through the production of ROS. Additionally, FFAs-induced NETs are capable of activating DCs, leading to CD4+ T cell differentiation into Th1 and Th17 cells, which release IL-1β, IL-12, and TNFα [201]. Akin to the cellular contact result demonstrated by Lambert et al., NET-stimulated CD4+ primary T cells differentiated into Th1 and Th17 cells at a significantly higher rate when co-cultured with DCs compared to when CD4+ T cells were cultured alone [201]. Zhang et al. used erythromycin to inhibit NET formation, downregulate Th1 and Th17 cells, effectively ameliorating disease in their emphysema model [202]. 

NETs inducing Th17 differentiation has been shown to result in neutrophilia in severe asthma [203]. Parallel exposure of mice to aeroallergen and endotoxin during sensitization lead to the formation of NETs. These activated DCs in vitro to activate IL-17 production from naϊve CD4+ T cells [203], unlike the differentiation process in psoriasis discussed previously, where Th17 cells are derived from memory T cells.

## 5. Lymphocyte Extracellular Traps

### B Cells

Recently, other leukocytes including B cells, T cells, natural killer cells, and monocytes were reported to release mtDNA following stimulation with class C CpG oligodeoxynucleotides (CpG-C) and non-CpG-C, short, single-stranded synthetic DNA. This was independent of B cell antigen receptor, TLR9, STING, and absent in melanoma 2 (AIM2) pathways, and did not involve an oxidative burst or result in cell death [6]. CpG and non-CpG oligodeoxynucleosides of class C were shown to induce B cells to extrude mtDNA, akin to the mitochondrial NETs formed by neutrophils. These were distinct from NETs due to their lack of antibacterial properties, instead with the purpose of acting as immediate activators of type I IFN production [6]. Incubation of B cell mtDNA webs with peripheral blood mononuclear cells (PBMCs) induced the production of IFNα. Similar to the vital form of NETosis, the B cells remain viable following mtDNA release [6]. Arrieta et al. also characterized the release of DNA from B cells following treatment with serum from patients with SLE, Sjögren syndrome (SS), cryoglobulinaemia, and from one individual with cryoglobulinaemic vasculitis [7].

As part of the extracellular trap (ET) study conducted by Arrieta et al. discussed above, T cells were also found to release DNA following stimulation with serum from SLE patients [7]. CD4+ T cells were characterized by Costanza et al. to release histone-coated DNA fibres in a ROS-dependent manner following treatment with anti-CD3 and anti-CD28 antibodies [8]. The authors termed the extracellular DNA threads ‘T helper-released extracellular DNAs’ (THREDS). They subsequently showed that THREDS were able to induce IL-2, GM-CSF, IFNγ, and TNFα production from naϊve CD4+ T cells [8], amplifying the inflammatory response. As discussed previously in this review, NET deposits have been found in the CNS, as acknowledged by Costanza et al. in this study, following which they used the experimental autoimmune encephalomyelitis (EAE), a CD4+ T cell-mediated MS model to investigate the therapeutic potential of mitochondrial ROS blockade using SKQ1. Splenocytes from treated mice showed reduced production of proinflammatory cytokines, GM-CSF, IFNγ, and IL-17A compared to the control group. Furthermore, histopathological analysis revealed significantly less immune cell infiltrate and demyelination in the treatment group [8].

Koh et al. also used anti-CD3 and anti-CD28 antibodies as stimuli to show extrusion of ETs from CD8+ T cells, which they termed ‘lymphocyte extracellular traps’ (LETs) [9]. They used confocal time-lapse microscopy to show that the fibrous structures connected the LET-releasing cell to adjacent cells, followed by death of target cells via the delivery of cytolytic vesicles. The authors went on to investigate the involvement of LETs in tegumentary *leishmaniasis*, a parasitic disease where CD8+ T cells play a central role in pathogenesis [207,208,209], as previous studies have found NET deposits in *leishmaniasis* skin lesions [210,211]. Using confocal microscopy, they colocalized DNA strands with cytolytic vesicles and correlated disease progression with increased overall LETs [9].

## 6. Extracellular Traps Are No Longer the Strict Domain of Neutrophils

### 6.1. Eosinophils

Eosinophils are bone-derived innate immune cells that make up 1–6% of the circulating blood under normal homeostasis. In contrast to neutrophils, eosinophils are larger (12–17 µM in diameter) and are readily identifiable by large specific granules which stain with eosin (hence the name Eosin-ophil). The nucleus is bilobed and easily identifiable with electron microscopy but is often obscured by granules under the light microscope [212]. Eosinophils are largely recruited as a main line of defense against parasitic helminths and are heavily involved in allergic responses secreting preformed Th2 cytokines such as IL-4. Unique to eosinophils is the large crystalloid granules which are large and contain preformed proteins: Eosinophilic peroxidase (EPO), major basic protein (MPB), eosinophil-derived neurotoxin (EDN), as well as cytokines (IL-2, -3-, 4, -5, -6, -10, and -12, TNFα, IFNɣ to name a few from an exhaustive list).

DNA extracellular traps released from eosinophils were first described by Yousefi et al. in 2008 [213] where extracellular DNA fibers were fund to be “catapulted” after eosinophils were activated with either IL-5, or IFNɣ and subsequently stimulates with LPS or C5a. The DNA was of mitochondrial origin and did not result in the death of the eosinophils. This left the question: “is this preformed in granules” or of nuclear origin? DNA released from eosinophils was observed in under a second, and was more than twice the size of the diameter of the cell. No evidence of apoptotic cell death was observed (using either phosphatidylserine (PI) redistribution or caspase 3 activation), clearly distinguishing the ET release from other forms of cell death. The release appeared from peri nuclear structures rather than the nucleus itself indicating a probable mtDNA location as the source of DNA. PCR investigation confirmed that the DNA was of mitochondrial source rather than nuclear.

After this initial report by Yousefi et al., further reports on eosinophil extracellular traps were published and the term EETosis or EETs was coined to describe this type of eosinophil cell death. Two types have been described very similar to what is seen in neutrophils vital and suicidal.

#### 6.1.1. Vital EETs

Vital EETs release mitochondrial DNA studded with the protein MBP and ECP. This form of DNA release does not result in the death of the cell. As described previously above, mtDNA is released, there is no evidence of cytoskeleton remodelling as seen in NETs or in suicidal EET formation. However, what the two forms of DNA release do have in common is a dependence on the NADPH complex and ROS production. In asthma, EETs are significantly more evident than non-asthma patients [10]. The DNA observed was determined to most likely be of mitochondrial origin as mitochondrial ATP synthetase subunit 6 was found on the extracellular DNA eosinophil strands. Further evidence that this form of EET did not result in cell death was determined by TUNEL assays which determined that the eosinophils in asthmatic biopsies were still viable and the extracellular DNA combined with eosinophil proteins was generated by active live eosinophils.

#### 6.1.2. Suicidal EETs

**Figure 4:**
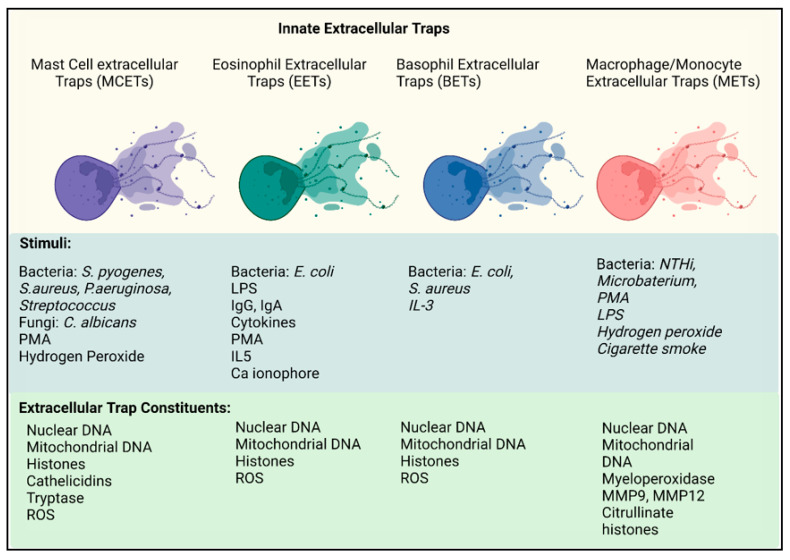
Innate extracellular traps. Mast cell extracellular traps (MCETs, purple) are stimulated by a range of pathogens including bacteria, fungi, PMA, and hydrogen peroxide. Once formed, MCETs consist of both nuclear DNA, mitochondrial DNA, histones and mast cell specific granules, tryptase, and chymase. Eosinophil extracellular traps (EETS, green) are simulated by bacteria, LPS, immobilized IgG and IgA, cytokines, IL-5, PMA, and Calcium ionophore. EETs are composed of DNA from both nuclear and mitochondrial origin, containing histones and ROS. Basophil extracellular traps (BETS, blue) are activated by bacteria and the cytokine IL-3. NET constituents are similar to that of EETS. Macrophage/Monocyte (METs, red) are activated by bacteria, PMA, hydrogen peroxide, and cigarette smoke. The webs contain nuclear DNA, myeloperoxidase, proteases: MMP9 and MMP12 and citrullinated histones. Figure created in Biorender.

The first report of EETs that resulted in the death of the cell was in 2013 by Ueke et al. [214]. Human eosinophils stimulated with either IgG, IgA, PMA, GM-CSF (+Platelet activating Factor (PAF), A23187, and IL5 +PAF all resulted in a lytic form of eosinophil cell death. EETs were clearly distinguishable from other forms of cell death such as necroptosis and apoptosis as no filamentous DNA was released. To determine if neutrophils EETs formation relied on NADPH oxidase, eosinophils were stimulated in the presence of the NADPH oxidase inhibitor DPI. No EETs were observed as a result, confirming EET formation is NADPH dependent. It is likely that the difference in the types of DNA release is related to the stimulus, some stimuli may activate pathways that promote the release of mtDNA, whereas other stimuli promote cell death (Figure 4).

#### 6.1.3. Are EETs Protective or Pathogenic?

EETs provide protection against fungi and bacteria by ensnaring pathogens in their large filamentous webs. Il-5 transgenic mice constitutively express IL-5, and results in excessive recruitment of blood eosinophils (40% of leukocytes compare to homeostatic conditions of less than 2%). Yousefi et al. used these mice to induce sepsis via a caecal ligation puncture (CLP) model, where eosinophils were recruited rapidly to sites of intestinal inflammation whereby they release EETs [213]. This phenomenon requires eosinophils to be activated by IL-5 as the WT mice had little evidence of eosinophilia or ET formation. The IL-5 transgenic mice were protected over time after CLP, with a significant reduction in proinflammatory cytokines and bacterial burden. This provided proof of concept data that eosinophils may be required to “plug” up breaches in the intestinal barrier thereby providing protection against sepsis. However, this could be a double-edged sword, and the recruitment of eosinophils may also induce inflammation. In inflammatory bowel disease (IBD) eosinophils are proinflammatory, where they contribute to diarrhea (as a promotility agent), contribute to tissue pathology in particular damage of the epithelial cells, and promote fibrosis [215].

The role of eosinophils in allergy and airway diseases are well known. Severe eosinophilic asthma (SEA), acute asthma, chronic eosinophilic rhinosinusitis (ECRS), and eosinophilic otitis media (EOM) are characterized by eosinophil infiltration and have been observed to have abundant EETs at sites of inflammation. 

In SEA, blood eosinophils stimulated ex vivo with either IL-5 or LPS had significantly more EETs when compared to patients with non-severe asthma (NSA). EETs behave in an autocrine fashion where they induce eosinophil granulation. Incubation of EETs remnants with eosinophils induced robust EET formation and increased ROS production [216]. EETs when co-cultured with epithelial cells (A549 cells, lung carcinoma epithelia cell line) resulted in 10% of the epithelia detaching from the culture well surface, increased epithelial cell permeability and the production of IL-8 and IL-6 release for the epithelial cells. This is a strong indication that the granules released in EETs are involved in perpetuating the airway inflammation observed is SEA patients [216].

In an animal model of acute asthma, EETs have been observed in bronchoalveolar lavage fluid (BALF). DNase I treatment in mice with induced asthma (induced by subcutaneous sensitization with OVA and subsequent intranasal challenge with OVA), reduced EETs and decreased airway resistance significantly [217]. It cannot be discounted that the removal of the mucus in the airways by DNase I, rather than the EET degradation accounted for the protective effect in this model of disease. DNase I is currently used to treat patients with cystic fibrosis to remove the mucous in the airways [218]. More work is needed to clarify if the DNase I is specifically clearing the observed EETs.

EETs have been found as predominant components of exudate from patients with ECRS and EOM [219,220]. In chronic eosinophilic rhinosinusitis (ECRS) it is proposed that nasal carriage of *Staphylococcus aureus* is inhibited by EETs which release mitochondrial DNA and ensnare the bacteria. In co-cultures *S. aureus* is able to induce EETs and prevent its growth [221]. Migration assays demonstrated that EETs migrate towards *S. aureus* and in particular migrate towards areas of epithelial disruption where *S. aureus* was trapped. Those patients with high levels of IL-5 in tissue had significantly more EETs present. These results suggest that eosinophils are recruited to sites of epithelial damage to provide host protection from *S. aureus.* It is possible that EETs may be required to maintain barrier function, to prevent pathological bacterial infections.

Eosinophilic otis media (EOM) is a middle ear disease characterized by eosinophils and eosinophilic mucus within the middle ear mucosa which causes hearing impediment. It is refractive to most treatment with the exception of steroid treatment which has large undesirable side effects [222]. A recent study has shown the EETs are a major feature of middle ear effusions, staining for EETs demonstrated that they were present in all patients. Combined with clinical evidence of high IL-5 cytokine production in these patients indicates that IL-5 released from eosinophils, recruits more eosinophils which ET and contribute to the viscosity of the mucus [222].

EETs have recently been shown to have a role in the pathogenesis of ANCA-associated vasculitis (AAV) [223]. In a study of 35 patients with AAV ([10] EGPA, [13] MPA, [12] GPA), those patients with EGPA only had significant serum cell free nuclear DNA and cell free mitochondrial DNA that associated with disease activity. In particular, levels of cf-DNA and cf-mtDNA were associated with the Birmingham Vasculitis Activity Score (BVAS is an assessment tool which measures disease activity in patients). EETs were observed around small-vessel thrombi in skin samples, and found occluding blood vessels. Eosinophils isolated from the blood were stimulated to form ETs in vitro and compared to NET formation by neutrophils. In comparison to NETs, ETs had condensed chromatin threads rather than the decondensed chromatin observed in NETs with an increased structural stability. DNase I was able to dissolve NETs readily whereas the stable structure of the ETs protected them from DNase I treatment. Little is known about the pathogenesis of EGPA and these observations indicated that therapy-targeting eosinophils and EETs may be of therapeutic benefit for patients, for example an IL-5 antibody treatment.

### 6.2. Mast Cells

Paul Ehrlich and Elie Mitchnikoff received the Nobel Prize over a century ago in recognition of their contribution to the field of immunology. Mast cells (MCs) were first described in Ehrlichs doctoral thesis who named them “Mastzellen” cells, “mast” in German means fattening, and Ehrlich thought the role of mast cells was to nourish the surrounding tissue through the release of the large granules. Elrichs discovered that mast cell granules specifically reacted metochromatically to aniline dyes, one of the methods still used to identify mast cells today. Elrich observed the presence of mast cells in chronic inflammation and tumors, and concluded they were there to nourish the tissue [224]. This we now know to be incorrect, but the actual function of mast cells eluded many scientists after Elrich, and to this day mast cells have been shown to be multi-faceted. They play a traditional role in innate immunity (helminth parasites), a role in allergy, and more recently have been shown to have immunomodulatory functions.

MCs are derived from CD34+/c-kit+/FcεRI-pluripotent progenitor cells. MCs have a long life, and are found in many organs throughout the body, but are most commonly found in skin and mucosal surfaces. MCs only mature once they are recruited into tissue, where they have the capacity to develop into a heterogeneous population. MC plasticity has recently been attributed to the diverse transcriptional properties of mast cells, which are distinct from other lymphoid and myeloid cell populations. Expression profiling of MCs, demonstrated that MCs are enriched in transcriptions for genes for a wide variety of proteases, sensing genes, and genes involved in metabolic pathways. Genes between the different tissue types of MCs differed widely, evidence that MCs can respond to their environment [225]. Once recruited from circulating blood into the tissues, MCs differentiate into different types of mast cells based on their location and granule content. In humans, MCs develop into three different subtypes: mast cells that are positive for tryptase (MCT), chymase (MCc), and double positive for both tryptase and chymase (MCTC) [226]. In mice, MCs are subtyped according to their location and heparin content within their granules. Connective tissue MCs (CTMC) which contain ample heparin-containing granules and mucosal mast cells (MMCs) where heparin is either absent or minimal [227].

The role of MCs in allergy has been well studied, especially in IgE-mediated allergic disease. Mast cell activation in the context of allergy, is regulated through FcεRI a high-affinity receptor for IgE [228]. Antigen-specific IgE is produced by B cells in response to antigen presentation by DCs. IgE binds to FcεRI on the MC surface, activating the cell to degranulate, which releases a multitude of different proteases (chymase, tryptase), histamine, serotonin, heparin, and secrete cytokines, all within minutes of exposure to the antigen [229].

MCs now recognized to play a role in adaptive immune responses. In vitro experiments on bone marrow-derived mast cells BMMC, demonstrated that TLR4 engagement of MCs induces the secretion of cytokines and chemokines cytokines such as IFNγ, IL-6, IL-4, IL13, TNFα, IL-5, instead of the release of histamine as TLR2 elicits [230]. These MC-derived cytokines can influence the polarization and activation of T cell subsets. CD4+ T cell effector functions can be regulated by MCs. IFNγ primed MCs under inflammatory conditions in human psoriatic skin can present antigen to CD4+ memory T cells, and skew the T cell response to an IL-22 response. MCs are often observed in close contact with T cells in psoriatic skin, where they are well placed to perpetuate inflammation [231]. 

#### Mast Cell Extracellular Traps

Mast cell extracellular traps (MCETs) can be triggered by a wide variety of stimuli, but they are very similar to what induces NETs, namely PMA, hydrogen peroxide (H_2_O_2_) and in response to several different pathogens including bacteria, protozoa, and fungi [232]. Similar to NETs, MCETS contain long strands of DNA decorated with mast cell granules, many of which have antibactericidal killing capability such as β defensins, LL-37, and piscidins [232] (Figure 4). Similar to neutrophils, they also require ROS-dependent mechanisms. 

In the context of infections, mast cells are often the first responders that release enzymes that recruit other immune cells such as neutrophils and macrophages. The first description of MCETs was in the context of *Streptococcus pyogenes* an opportunistic bacterium that causes major skin infections such as impetigo and necrotizing fasciitis. In this study, it was observed that mast cells were able to restrict the growth of bacteria independent of phagocytosis [233]. Regardless of whether the mast cell was of human or murine origin, incubation with *Streptococcus pyogenes* induced MCET formation, and *Streptococcus pyogenes* was observed trapped in the filamentous DNA extruded from the mast cells. The entrapment *of Streptococcus pyogenes* within the DNA killed the bacteria (observed by live/dead stain). Unlike NETs which can be inhibited with DNase I, MCETs required co-incubation with both DNase I and myeloperoxidase (MPO) to inhibit ET formation. The addition of MPO targets the tryptase within the NETs as it is a potent inhibitor of mast cell tryptase [234].

MCETs also inhibit the growth of *Staphylococcus aureus* in the initial phase of infection. *Staphylococcus aureus* causes skin infections, pneumonia, and can induce septicemia [235]. *Staphylococcus aureus* strains have developed that are resistant to treatment via antibiotics; therefore, understanding mechanisms in which the host can defeat this pathogen are important. In the case of mast cells in *Staphylococcus aureus* infection, they are both protective and pathogenic during an infection. In protection, mast cells release MCETs which have an antimicrobial effect killing the bacteria they trap both within the DNA and via release of antimicrobial compounds released from mast cell granules. The pathogenic effect is that *Staphylococcus aureus* is able to internalize within the mast cells in a subterfuge manner where it avoids detection and elimination. Live *Staphylococcus aureus* can be found viable up to 5 days later within mast cells [235].

MCET formation is stimuli dependent [236]. DNA release from mast cells can be independent of ROS production depending on the response of mast cells to different pathogens. ROS production and extracellular DNA release measured via FACS analysis has shown that both L. Monocytogenes and S. aureus can release DNA independent of ROS production [236]. It is possible that this observation may be similar to what is seen in other leukocyte cell populations and may be a form of “vital” release of extracellular DNA early on in exposure to the pathogen. Late exposure of *L. Monocytogenes* to mast cells can also induce “suicidal” ET release.

*Group A streptococcus* (GAS) causes skin infections, glomerulonephritis (acute kidney injury), pharyngitis, sepsis, and endocarditis [237]. GAS can also induce MCET formation, mediated through M1 protein. M1 protein is a critical virulence factor that contributes to the pathogenicity of GAS. M protein promotes the survival of GAS and replication by interacting with serum proteins (C3b) within human blood blocking opsonization of the bacteria. M1 mutant mice have significantly reduced capacity to produce NET and MCETs, suggesting that M1 alone is enough to induce MCET formation [237].

*C. albicans* is one of the most common opportunistic fungal infections in humans. In homeostasis conditions, *C. albicans* is a commensal which inhabits in skin and mucosal surfaces (urogenital, gastrointestinal, and the oral cavity). Immunosuppressed patients in particular are at risk as *C. albicans* can invade noncommensal tissue and manifest a pathogenic response in tissue. Mast cells which are predominant in mucosal surfaces are the first innate cell to come into contact with *C. albicans* where degranulation can inhibit and restrict the growth of *C. albicans* [238]. *C. albicans* can also stimulate MCET formation but not inhibit the growth. After 6 h incubations with *C. albicans,* mast cells release extracellular DNA in a suicidal fashion, with the release of nuclear DNA, histones, and enzymes. However, this does little to inhibit the growth of the fungus, the reduction in *C. albicans* is actually attributed to the release of mast cell granules. The only function of MCETs in the context of *C. albicans* infection is in providing a physical barrier in which the fungus is trapped.

MCETs may play a role in limiting bacterial growth but also have a dark side. In inflammatory conditions such as psoriasis, mast cells that release MCETs also release large quantities of IL-17a, a proinflammatory cytokine which recruits neutrophils [199]. Mast cells within the papillary dermis of psoriasis plaques not only degranulate but also form MCETs, with large strands of extracellular DNA containing large quantities of IL-17. In an ex vivo model of normal human skin explants IL-1β and IL-23 can induce MCET formation [199]. This suggests that in inflammatory conditions, the release of proinflammatory cytokines from damaged epithelial cells and leukocytes recruited to the skin (e.g., T cells and neutrophils) has the potential to activate tissue-resident mast cells to form MCETs, perpetuating the vicious cycle of inflammation.

MCETs are associated with the pathogenic progression of thrombus formation in patients with thrombotic complications after myocardial infarction [239]. Autopsy material of the thrombus from patients has significant numbers of NETs, EETs, METs, and MCETs. Although the number of NETs and METs outweigh the number of EETs and MCETs. MCETs observed within the plaques is of concern even in small numbers as they have the power to destabilize the plaque through the release of proinflammatory cytokines and the release of enzymes, in particular mast cell-specific histamine, chymase, and tryptase [239].

### 6.3. Basophils

Basophils comprise less than 0.5% of the white blood cells and are largely associated with playing a role in allergic diseases and in response to infection with parasites. Similar to mast cells they share the unique ability to release inflammatory cytokines and enzymes that instigate inflammation in allergic inflammation [240]. The function of basophils is controlled by the cytokine IL-3, it promotes both differentiation and survival and primes basophils to release leukotriene C4. Priming basophils with IL-3 can stimulate basophil extracellular traps (BETs) in a concentration-dependent manner. This requires crosslinking of the high-affinity IgE receptors. If these receptors are blocked, no BET formation is observed. After crosslinking IgE receptors, basophil mitochondria and granules track toward the outer cell membrane. The nucleus stays intact, and histones are not observed on the extruded DNA indicating it is likely to be of mitochondrial origin and not nuclear. This is a NADPH ROS-independent mechanism as basophils lack components critical for the NADPH complex (p47 phox and p67 phox). Instead, mitochondrial ROS provides the energy required for DNA release. Further evidence of mtROS instigating the process is that MitoQ, an inhibitor of mtROS, prevents the formation of BETs.

Both *E. coli* and *S. aureus* also stimulate BET release, through a NADPH-independent mechanism. Basophils co-cultured with both *E. coli* and *S. aureus* stimulate the release of mtDNA whilst the nuclear DNA stays intact within the cell. *E. coli* and *S. aureus* trapped with the DNA of BETs are actively killed, suggesting that BET formation is a further mechanism for host defense against bacterial infection [13] (Figure 4).

### 6.4. Monocytes/Macrophages

Monocytes are generated from hematopoietic stem cells in the bone marrow. Monocytes migrate from the circulating blood stream into the tissues where they differentiate into macrophages. The primary function of macrophages is host defense, through phagocytosis of microorganisms, clear cell debris from cell death (apoptosis, NETs, necrotic cells, etc.) and initiate tissue repair and healing. Macrophages are also able to present antigen to T helper cells via MHC class II molecules. In inflammation macrophages can be divided into either M1 or M2 macrophages based on their phenotype. M1 macrophages (also known as classically activated macrophages) are generated in response to IFNγ and LPS and are considered to be proinflammatory as they secrete IL-12 and IL-23. M2 macrophages (alternative macrophage activation) are induced by IL-4 and IL-13, and preferentially secrete IL-10 over IL-12.

#### Macrophage Extracellular Traps (METs)

*Haemophilus influenzae* (NTHi) is a commensal bacteria present in the pharynx of adults in normal homeostatic conditions. However, NTHi can move further down the airways and take up residence in the lower respiratory tract in patients with chronic bronchitis and chronic obstructive airway disease (COPD) [241]. Macrophages from patient bronchial alveolar lavage (BAL) fluid samples produce ROS when stimulated with NTHi and extrude large nuclear extracellular DNA strands of DNA similar to what is seen in NETs. These structures termed macrophage extracellular traps (METs) are decorated with MMP12, and citrullinated histones. The release of proteases by METs have the potential to exacerbate conditions such as emphysema where MMP12 has already been established to play a role in the progression of disease [241]. In vitro NETs and METs from BAL fluid can be inhibited with DNase I, suggesting a possible therapeutic avenue for treatment for patients.

Cigarette smoke is a critical cause of lung disease, playing a role in chronic inflammation which can manifest clinically as emphysema and COPD. Cigarette smoke causes a sustained production of proteases and exhausts the endogenous supply of alpha-1 antitrypsin (AAT) the natural inhibitor of neutrophil elastase [242]. Cigarette smoke induces alveolar MET formation in vitro where they express both MMP9 and MMP12 which colocalize with extracellular DNA citrullinated histones and PAD2. In contrast to NETs, alveolar METs in the study extruded shorter strands of DNA but similar to NETs had high expression of ROS. Using a cigarette smoke mouse model, the number of macrophages recruited to the lungs was 2-fold that of mice not exposed to cigarette smoke. Mice exposed to cigarette smoke and treated with DNase I had significantly less macrophages, whereas DNase I had little effect on the recruitment of neutrophils. Exposure to cigarette smoke resulted in 5 times the amount of production of METs when compared to NETs. Suggesting that macrophages are more sensitive to activation by cigarettes smoke than neutrophils.

Children experience frequent respiratory infections which may be detrimental to the development of healthy lungs. Children unlike adults are not as effective at clearing these infections. BAL samples from 76 pediatric patients with lung disease (38 cystic fibrosis and 38 non-cystic fibrosis subjects) were examined ex vivo for extracellular trap formation. NETs were more prevalent than MET formation; however, stimulation with NTHi significantly increased the numbers of both NETs and METs [243]. Combination of both DNase I and alpha-1 antitrypsin (AAT) produced a significant reduction in the number of NETs and METs (20× fold) compared to either treatment used alone. Interestingly, DNase I alone increased neutrophil elastase activity, presumably as NE is protected within the DNA strands of extracellular traps, which is released when DNase I is added.

*Escherichia coli* and *Candida albicans* induce MET-like structures in J774A.1 macrophage cell lines and peritoneal lavages [244]. METs contained both nuclear and mtDNA and were decorated with enzymes and histones. MET formation was independent of ROS produced by NADPH and had limited ability to kill the bacteria (55% effective) or fungus (18%) ensnared in their traps (Figure 4). Additional stimuli were used to induce MET formation such as LPS, PMA, and H_2_O_2_ which are potent NET stimulators; however, they failed to induce METs. This is in direct conflict with other reports that have shown MET formation with these stimuli and that MET production is dependent on NADPH ROS [11,12]. This could be due to the type of cells being used which may not be as effective at inducing MET formation, with one being a cell line and the other cells from peritoneal lavage fluid. 

The first description of macrophage extracellular traps (METs) in autoimmune disease was the observation of (METs) within the kidney glomeruli of patients with MPO-ANCA-associated vasculitis [149]. Macrophages are the predominant cell type within kidney biopsies. MPO the autoantigen in this disease is thought to of been released mainly by neutrophils either through degranulation or the production of NETs. This study demonstrated that there were equal numbers of MPO-positive macrophages compared to MPO-positive neutrophils, suggesting that macrophages, and subsequent METs may be an overlooked additional mechanism by which MPO is deposited in the kidney.

Similar to NETs, there are still many discrepancies in the literature regarding the formation of METs. This could also be due to different types of cell death with those being NADPH ROS dependent being “suicidal” MET formation and those independent of NADPH ROS being “vital” relying on mtROS. There are still studies that need to be conducted to examine if different subtypes of macrophages are more susceptible to forming METs than other cell types (e.g., M1 vs. M2). The pathways that lead to MET formation have not been as well characterized as that of NET formation, with many questions remaining on what drives the macrophage to MET formation.

### 6.5. Dendritic Cells

Dendritic cells (DCs) are professional antigen-presenting cells (APCs), derived from hematopoietic bone marrow progenitor cells. Their primary function is to present antigen proteins from microorganisms to T cells. Immature DCs reside in the epithelia such as skin, whereas mature DCs reside in the lymph nodes and spleen. DCs can be divided further into two subsets, known as classical and plasmacytoid. Classical DCs are present within epithelia, peripheral tissue, and lymphoid organs. Plasmacytoid DCs are present both in the tissue and blood and they are the primary producers of type 1 interferons.

Very few data are available on the formation of dendritic cell extracellular traps. One report describes the release of extracellular DNA by plasmacytoid DCs after incubation with A. fumigatus [245]. After a 4 or 6 h incubation of pDCs with *A. fumigatus* extracellular DNA could be observed containing citrullinated histones and spread over the *A. fumigatus* area. The percentage of DC ETs was approximately 1% which is comparable to other studies that show neutrophils NET in response to other fungi (*Candida albicans*) at the same rate [246]. The study does not examine if the release of DNA inhibits or kills *A. fumigatus*. More work needs to be carried out to see if this response is detrimental to the host or if it functions as a critical part of host protection.

## 7. Potential Therapy Targeting the Injurious Functions of Extracellular Traps

The evidence that NET/ETs can drive proinflammatory pathologies is extensive, consequently they are promising targets for new specific alternative therapy to broad spectrum immunosuppressive agents. Current immunosuppressive agents such as cyclophosphamide and broad-spectrum corticosteroids are associated with adverse side effects and have no specificity. The advantage of most of the inhibitors of ETs is that they are reversible and can be temporarily withdrawn if patients contract an infection that require ETs to efficiently clear it. In contrast, current conventional treatment if halted can take weeks to months before the immune system is restored.

All the leukocyte extracellular traps described within this review have different mechanisms of action and effectiveness against different human pathogens; however, they all have similar pathways to extracellular trap formation providing different avenues of possible therapeutic strategies. (1) Target the extracellular traps themselves once expelled by digesting the ET remnants (DNase I). (2) Target enzymes required for digesting either the nuclear envelope, outer cell membrane, or citrullination of histones (PAD family, NE, MPO, GASDERMIN D). Each of these distinct critical components will be considered and discussed briefly as more diverse treatment options have been extensively reviewed recently [247,248].

### 7.1. Therapy That Targets Extracellular Traps after Formation

#### DNase I

DNase I is the most commonly used enzyme to disrupt NETs both in vivo (in animal models of inflammatory diseases) and in vitro. It is a safe choice as it is already used clinically to treat patients with cystic fibrosis, and is well tolerated with few side effects, so is readily poised for translation to other diseases [218,249]. DNase I belongs to a large family of DNases comprising DNase I, DNase 1L1, DNase 1L2, and DNase IL3 [250]. DNase I and DNase L3 both show potential in targeting ET-driven pathologies. The two enzymes work synergistically, with DNase I targeting double-stranded DNA (dsDNA) and extracellular nuclear proteins. DNase 1L3 plays more of role in the degradation of intracellular DNA, nucleosomes, and DNA protein complexes [251]. Both enzymes degrade NETs, and there are multiple reports on DNase I disrupting METs, MCETs, and EETs already reviewed in the previous section.

The major issue with DNase I as a therapy is its relatively short half-life of 3–4 h, and its rapid inactivation by G actin in the circulation. Multiple patents are already in place to protect research developing actin-resistant DNase I for the treatment of cystic fibrosis to combat this issue (NCT02605590, NCT02722122). Other avenues to increase the half-life of DNase I are experimental, using cutting edge technology. Gene delivery vectors have already been used in animal models of sepsis where plasmid expression of both DNase I and DNase 1L3 have been shown to maintain stable therapeutic levels of DNase [251].

### 7.2. Therapeutic Targeting of Critical Enzymes Required for ET Formation

#### 7.2.1. Peptidyl Arginase Deiminases

PAD4 is a critical enzyme that citrullinates histones by converting a positive arginine to a negative citrulline residue. This has a dramatic effect on cell signaling, inducing chromatin decondensation which facilitates the expulsion of nuclear DNA [252]. There are 5 different PAD enzymes, PAD 1, 2, and 4 are expressed in the immune system, as well as other tissue whereas 1, 3, and 6 are expressed in tissue only. A common requirement of all PAD enzymes is calcium-dependent catalysis, where intracellular calcium concentrations have to rise above homeostatic conditions to promote activity. PAD4 is largely associated with the production of NETs, and is less observed in other forms of ET formation. PAD2 has been associated with MET formation. The role of PAD expression is not well defined in EETs, MCETs, and BETs.

Therapeutic targeting of PAD4 has been well explored in many inflammatory conditions. The main therapeutic focus is on various forms of chlorine amidine which inactivates all the PAD isoforms. Cl-amidine and BB-Chlorine amidine have been explored extensively in the context of autoimmune kidney diseases, showing protective effects in lupus, where it inhibits NET formation and reduces proteinuria (clinical indicator of renal function) whilst also protecting against skin inflammation [253,254]. Therapeutic use of Cl-amidine also provides protection in a model of murine sepsis, where overall survival is improved through the inhibition of NETs [255]. In murine atherosclerosis Cl-amidine can reduce vascular damage and modulate innate immune responses (through a reduction in macrophages in arteries) [256].

Inhibition of PAD4 is not without concern. Citrullination of histones in NET formation is not the only physiological function it performs. PAD enzymes also play a role in gene regulation and cell differentiation [257]. Inhibition of PAD4 is also deleterious in certain forms of bacterial infection. PAD4−/− mice are more susceptible to infection in models of necrotizing fasciitis, as NETs are required to efficiently clear *S. flexneri* and *Group A Streptococcus* (GAS). Pathology in PAD4−/− mice infected with GAS was quadruple of that seen in WT mice [55].

#### 7.2.2. Neutrophil Elastase Inhibition

A promising therapeutic avenue is the inhibition of neutrophil elastase (NE). NE plays several different roles in the formation of NETs. Firstly, it migrates to the nuclear envelope where it helps break down the nuclear membrane and cleaves histones. Secondly, NE cleaves GSDMD activating it to create pores in the outer cell membrane to allow expulsion of nuclear DNA. Inhibiting any of these functions will inhibit NET formation. In terms of the proinflammatory role NETs play, the DNA backbone of the NET shields NE from endogenous inhibitors providing an injurious reservoir of proteases that directly injure tissue.

NE inhibitors are already well-developed clinically with multiple clinical trials in various stages for treatment of inflammatory lung diseases such as cystic fibrosis, COPD, and bronchiectasis [258,259,260]. Both Alvelestat (AZD9668) and BAY 85–8501 are the leading candidates, they are both reversible and have similar potency to that of endogenous antiproteases. Importantly both have been shown to inhibit NETs in vivo and in vitro [261].

#### 7.2.3. Gasdermin

As mentioned previously, Sollberger et al. [59] demonstrated that neutrophil elastase cleaves GSDMD, which punches holes in the nuclear envelope and outer cell membrane to facilitate the release of extracellular DNA. Using a chemical screen to identify potential inhibitors of NETosis, the authors found a compound that both inhibits the inflammasome and NET formation—LDC7599. Through elegantly designed experiments Sollberger et al. demonstrated that GSDMD was required for NETosis (using GSDMD mutant mice where NET formation was significantly abrogated). LDC7599 is still clinically an experimental drug, but shows therapeutic potential.

Disulfiram is currently used therapeutically to treat alcoholism. Disulfiram inhibits aldehyde dehydrogenase irreversibly preventing ethanol conversion into acetaldehyde, which causes adverse effects when taken with alcohol causing nausea and tachycardia powerful deterrents to reduce alcohol consumption. Hu et al. [262] discovered, using a high-throughput biochemical screen, that disulfiram can inhibit GSDMD pore formation. Therefore, it stands to reason it will also block NET formation. 

As already discussed, NET formation is a prominent feature of lung pathology in the SAS-CoV-2 infection. Using a rodent infection model of SARS-CoV-2, it has been shown that disulfiram can significantly reduce NET formation and increase survival demonstrating its therapeutic potential [263]. As disulfiram is FDA approved and is well-tolerated clinically, it is well poised for translation to treat NET-driven pathologies. A clinical trial has already commenced to evaluate the efficacy of disulfiram in treating patients with moderate COVID-19 (NCT04594343).

## 8. Conclusions

Extracellular trap formation from innate immune cells has likely evolved to enhance host defense. Historically, humans prior to the industrialization of the world and modern medicine would have needed extracellular traps to help reduce infection. Akin to the redundancy of an appendix, this innate mechanism is a vestigial hangover from a time when living conditions and modern medicine were lacking. The evidence suggests that extracellular traps are now more harmful contributing to inflammation and pathology in a range of diseases. In autoimmunity, NETs alone release over 70 known autoantigens. Given the fact that many current treatments for inflammatory diseases are non-specific, targeting NETs/ETs may provide a specific treatment to reduce the burden of disease with minimal side effects. This expanding field of cell death and immunology still has questions left unanswered. It is still unknown why a leukocyte is driven down the pathway of extracellular traps or why some stimuli are potent stimulators of extracellular traps and others are not. 

No clinically approved specific therapy to target NETs/ETs currently exists. The majority of the inhibitors discussed here are already used clinically to treat other inflammatory diseases so are reasonable candidates to put forward for treatment of diseases where extracellular traps are prominent features of the pathology.

## Data Availability

Not applicable.
